# The mechanism of genome replication and transcription in bunyaviruses

**DOI:** 10.1371/journal.ppat.1011060

**Published:** 2023-01-12

**Authors:** Hélène Malet, Harry M. Williams, Stephen Cusack, Maria Rosenthal

**Affiliations:** 1 University Grenoble Alpes, CNRS, CEA, IBS, Grenoble, France; 2 Institut Universitaire de France (IUF), Paris, France; 3 Bernhard Nocht Institute for Tropical Medicine (BNITM), Hamburg, Germany; 4 Centre for Structural Systems Biology, Hamburg, Germany; 5 European Molecular Biology Laboratory, Grenoble, France; 6 Fraunhofer Institute for Translational Medicine and Pharmacology (ITMP), Discovery Research ScreeningPort, Hamburg, Germany; Boston University, UNITED STATES

## Abstract

Bunyaviruses are negative sense, single-strand RNA viruses that infect a wide range of vertebrate, invertebrate and plant hosts. WHO lists three bunyavirus diseases as priority diseases requiring urgent development of medical countermeasures highlighting their high epidemic potential. While the viral large (L) protein containing the RNA-dependent RNA polymerase is a key enzyme in the viral replication cycle and therefore a suitable drug target, our knowledge on the structure and activities of this multifunctional protein has, until recently, been very limited. However, in the last few years, facilitated by the technical advances in the field of cryogenic electron microscopy, many structures of bunyavirus L proteins have been solved. These structures significantly enhance our mechanistic understanding of bunyavirus genome replication and transcription processes and highlight differences and commonalities between the L proteins of different bunyavirus families. Here, we provide a review of our current understanding of genome replication and transcription in bunyaviruses with a focus on the viral L protein. Further, we compare within bunyaviruses and with the related influenza virus polymerase complex and highlight open questions.

## Introduction

The order *Bunyavirales* constitutes a diverse group of predominantly insect or rodent-borne viruses with a segmented single-stranded RNA genome of negative polarity. In 2017, the formerly separated families of *Bunyaviridae* and *Arenaviridae* were combined to form the order of *Bunyavirales* [1,2], which currently includes 14 different families (https://talk.ictvonline.org/taxonomy/). Some bunyaviruses such as Lassa virus (LASV), Rift Valley fever virus (RVFV), and Crimean-Congo hemorrhagic fever virus (CCHFV) are of general public health concern as emphasized by their inclusion in the WHO R&D Blueprint list of priority diseases [3] because of their epidemic potential and the lack of specific medical countermeasures. However, many more bunyaviruses can cause severe human disease and even local outbreaks, such as La Crosse virus (LACV), severe fever with thrombocytopenia syndrome virus (SFTSV) as well as hantaviruses. Bunyaviruses additionally cause economic losses, for example, recurring outbreaks of RVFV in ruminants in Africa and the Arabian Peninsula are associated with abortions and death of livestock [4]. A comprehensive understanding of the viral amplification cycle and disease pathogenesis is needed to support development of effective treatments for bunyavirus infections. Here, we provide an overview of the current state of knowledge on the mechanism of bunyavirus genome replication and transcription, a key multistep process in the viral amplification cycle, centered on the large multidomain and multifunctional viral polymerase, called the L protein.

After attachment and entry of bunyaviruses into host cells, the viral RNA (vRNA) genome segments associated with the viral L protein and the nucleoprotein (NP) are released into the cytoplasm where viral transcription and genome replication take place. The 3′ and 5′ termini of each genome segment are highly conserved within each bunyavirus species. They are also highly complementary and thus capable of forming a double-stranded RNA (dsRNA) stem, allowing each genome segment to potentially form a pseudo-circularized panhandle [5,6]. However, in reality, the 3′ and 5′ termini are bound in specific sites on the L protein and partially form a distal duplex only when the polymerase is not actually performing RNA synthesis (see below). Genome transcription is initiated by a primer, derived by a so-called cap-snatching mechanism [7], and results in the production of capped, but usually non-polyadenylated messenger RNA (mRNA). Genome replication, however, is initiated *de novo* typically by a prime-and-realign mechanism and proceeds through a complementary positive-sense intermediate of the viral genomic RNA (cRNA) [8]. Some bunyaviruses, such as arenaviruses and phenuiviruses, use an ambisense coding strategy in which viral genes are encoded in opposite orientations on the same genome segment and separated by a highly structured intergenic region. Both the intergenic region and the 3′ and 5′ termini constitute *cis*-acting elements essential for genome replication and transcription processes.

**Fig 1:**
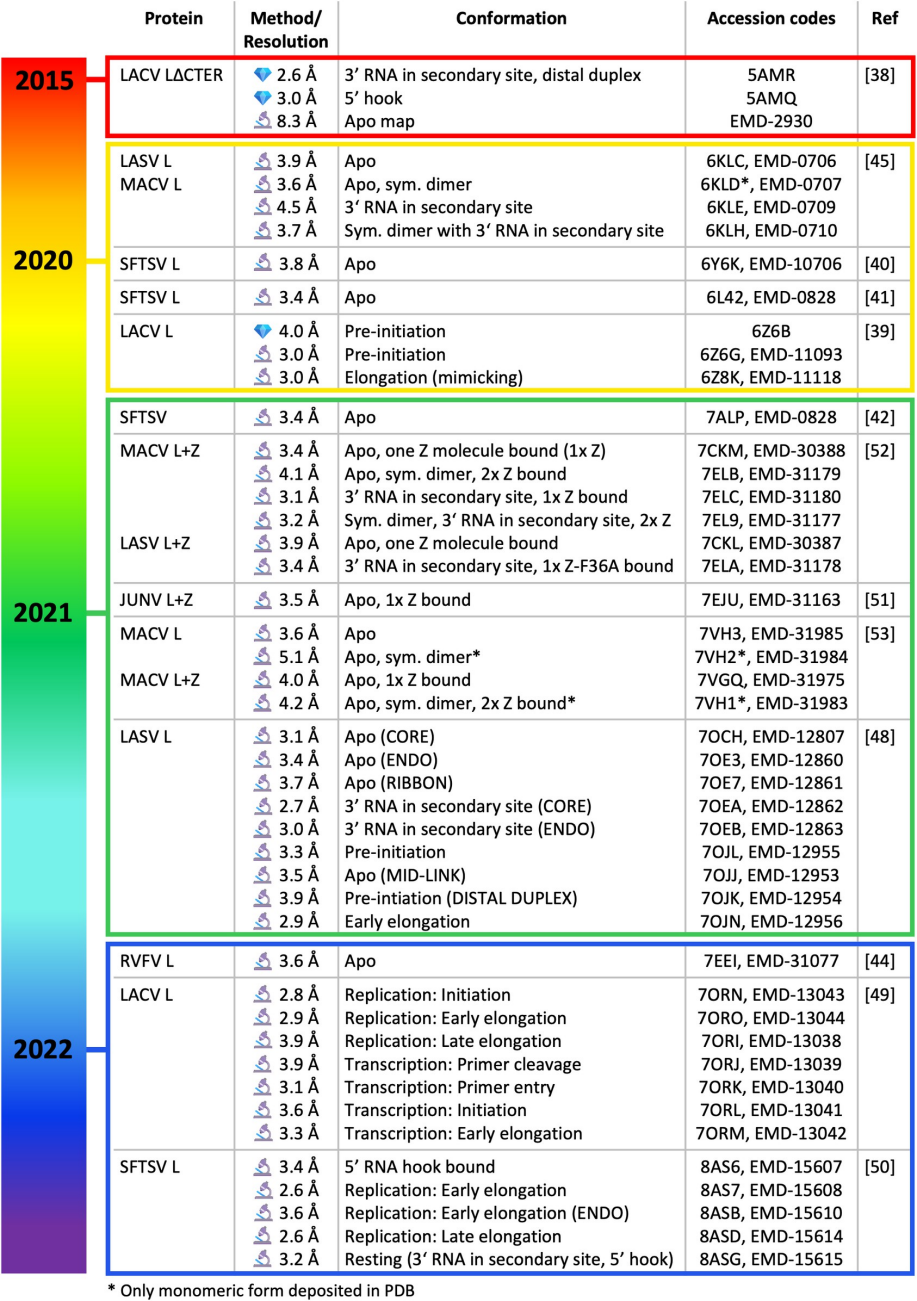
Overview of bunyavirus L protein structures. All published structures of bunyavirus L proteins are included in the table (JUNV, Junin virus; LACV, La Crosse virus; LASV, Lassa virus; MACV, Machupo virus; RVFV, Rift Valley fever virus; SFTSV, severe fever with thrombocytopenia syndrome virus). Some structures additionally include viral matrix protein Z (L+Z). Entries are organised firstly by year and then publication date. For each structure, the method of determination (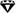 for X-ray crystallography, 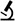 for cryo-EM) along with the calculated resolution, information on the protein conformational state (structures containing no viral RNA are marked as “apo”), accession codes for the Protein Data Bank (PDB) and/or Electron Microscopy Data Bank (EMDB) as well as the publication reference (Ref) are provided. The microscope and crystal emojis are open source from Microsoft (MIT license, https://github.com/microsoft/fluentui-emoji).

Although several bunyavirus NP structures have been published since 2010 [9–37], structures of L proteins from the *Bunyavirales* order were long awaited in the field and their appearance is summarized in Fig 1. The first L protein structure to be determined was that of LACV, from the *Peribunyaviridae* family, using a construct with a truncated C-terminus [38]. It was solved in 2015 in a pre-initiation state bound to its 5′ and 3′ promoters by X-ray crystallography and in an *apo* form by low-resolution cryogenic electron microscopy (cryo-EM). A high-resolution cryo-EM structure of full-length LACV L was subsequently determined in 2020, containing the C-terminal region (CTER) that was missing in earlier structures [39]. Concomitantly, high-resolution cryo-EM structures of L proteins from other bunyavirus families were determined, including SFTSV and RVFV L proteins *(Phenuiviridae*) [40–44], as well as Machupo virus (MACV) and LASV L proteins (*Arenaviridae*) [45]. However, arguably the major breakthrough in recent years has been the visualization of these proteins in action by determining structures of L proteins stalled in key active states. Following the pioneering work on influenza virus polymerase [46,47], snapshots have now been obtained for LACV, LASV, and the SFTSV L proteins [48–50]. LACV L structures revealed the key conformational changes necessary for genome replication and transcription [49]. SFTSV and LASV L snapshots depicted the conformational changes associated with promoter binding and genome replication activities [48,50] (Fig 1). In addition, LASV, Junin virus (JUNV), and MACV L have been determined in complex with the viral matrix protein Z, providing structural insights into the regulation of *Arenaviridae* polymerase activity [51–53], which had been detected previously in *in vitro* and cell-based assays [54–59]. Overall, these L protein structures allow differences and commonalities in the domain organization of bunyavirus L proteins to be identified and, in doing so, reveal the enormous flexibility of this multifunctional molecular machine. Here, we provide an overview of the recent scientific progress on the structural and functional understanding of bunyavirus genome replication and transcription processes. We compare structural and functional data between different bunyavirus families and identify current knowledge gaps.

### An overview on the structural organization of L protein

A striking observation when comparing the available bunyavirus L protein structures is that their architecture corresponds to a concatenation of PA, PB1, and PB2 subunits of influenza viruses. This is despite the fact that the influenza virus PB2 CTER shows very low sequence similarity with bunyavirus L proteins [7,60,61]. Furthermore, domains that are present only in some bunyavirus families such as an N-terminal ovarian tumor-like (OTU) domain for the nairoviruses and tenuiviruses [62–64] are missing in the influenza virus polymerase complex.

**Fig 2:**
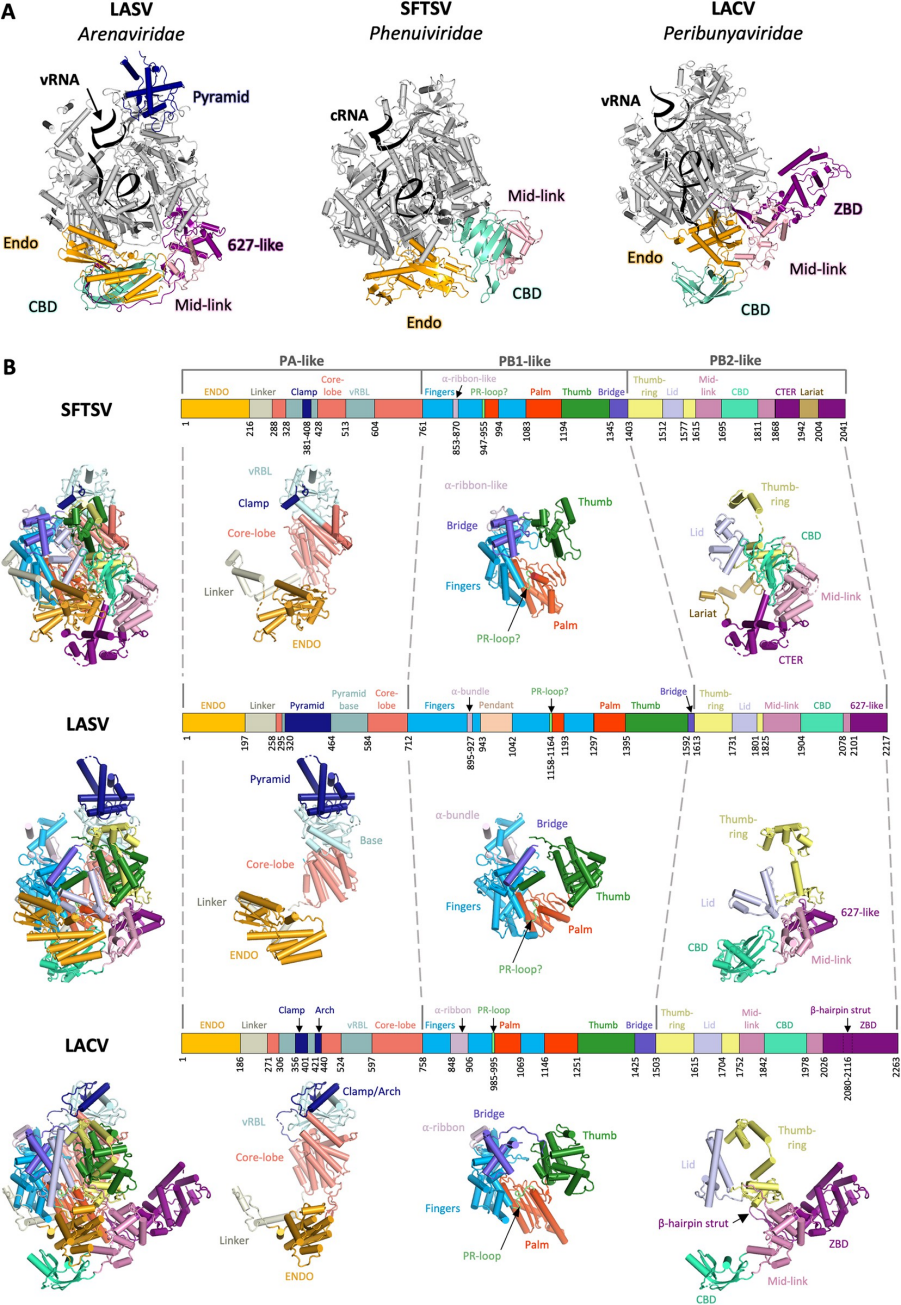
Structural comparison of LASV, SFTSV, and LACV L proteins. **(A)** The LASV late-elongation (PDB: 7OJN) [48], SFTSV late-elongation (PDB: 8ASD) [50], and LACV early-elongation (PDB: 7ORM) [49] L protein structures are shown side by side. The core of each L protein is shown in grey with key domains colored as follows: endonuclease (orange), pyramid domain (only in LASV, dark blue), CBD (cyan), mid-link domain (pink), and 627-like/zinc-binding domain (purple). Bound RNAs are shown as black ribbons. **(B)** The LASV late-elongation (PDB: 7OJN,) [48], SFTSV apo structure (PDB: 7ALP) [42], and LACV early-elongation (PDB: 7ORM) [49] structures are shown stacked in panels. Each panel contains a schematic linear representation of the L protein domain structure with the size of the individual domains scaled to represent their relative size in the full-length L protein. Individual domains are labelled and colored for clarity. The colors for each domain are mapped onto a cartoon representation of each L protein, which is then broken down into the influenza-corresponding PA-like, PB1-like, and PB2-like domains. Figures were created using CCP4mg [68] and Pymol (Schrödinger, LLC). CBD, cap-binding domain; CTER, C-terminal region; LACV, La Crosse virus; LASV, Lassa virus; SFTSV, severe fever with thrombocytopenia syndrome virus; vRBL, vRNA-binding lobe; vRNA, viral RNA; ZBD, zinc-binding domain ENDO, endonuclease domain.

In the bunyaviral L protein, the PA-like region contains (i) an endonuclease domain (ENDO) that undergoes large, rigid-body displacements during L protein activity; (ii) an elongated linker-region; and (iii) a PA-C-like domain that is defined as core lobe and vRNA-binding lobe (vRBL) in *Peribunyaviridae* and *Phenuiviridae* L proteins but which is known as the pyramid and base in *Arenaviridae* L proteins (Fig 2). Both the linker region and the PA-C-like region buttress the central PB1-like region, which contains the conserved palm, thumb, and finger domains, features that are present in all viral RNA-dependent RNA polymerases (RdRps) and form the RdRp active site. The PB2-like region contains (i) a thumb-ring that is homologous to PB2-N in influenza virus and which surrounds the thumb domain; (ii) a bridge that connects the thumb/thumb-ring to the fingers; (iii) a lid that plays a central role in separating the template from the product facilitating their exit as single-stranded RNAs; and (iv) a protruding, multidomain CTER that undergoes large rearrangements during activity and is not visible in all the experimental maps of full-length L proteins [39,48–50,65].

The globular L protein CORE is composed of the elongated PA-like linker, the PB1-like region, and the PB2-like lid. It contains a large central cavity that encompasses the RdRp active site including the canonical motifs A to F that are essential for RdRp activity. These motifs (i) select and stabilize the NTP that is complementary to the RNA template; (ii) coordinate two metal ions that are necessary for a nucleophilic attack on the α-phosphate of the fixed nucleotide; (iii) translocate the template and the nascent product after nucleotide incorporation; and (iv) reinitiate the catalytic cycle. Motifs A to E are localized in the palm whereas motif F is part of the fingers. Additional specific motifs of segmented negative-strand viruses (sNSV) include motif G in the thumb and H in the fingers domain [38,40,48,66]. The central active site cavity is connected to the exterior by four tunnels that correspond to (i) template entry; (ii) nucleotide entry; (iii) template exit; and (iv) product exit channels [8,38].The ENDO and the CTER protrude from the CORE and undergo large movements during activity [39,48–50].

The CTER displays the highest sequence divergence within the *Bunyavirales* and also in comparison to the PB2 protein of *Orthomyxoviridae* [60,67]. It contains a so-called mid-link domain that connects the CORE of the L protein to its CTER (Fig 2). Inserted into the mid-link domain is a cap-binding domain (CBD) or CBD-like domain as well as a variable C-terminal domain(s) (CTD) [7,48,49,60,61]. It is noteworthy that the position of the CBD and CBD-like domains does not appear to be strictly conserved within the sequence of the L protein (1842–1977 for LACV L; 1904–2077 for LASV L; 1695–1810 for SFTSV L) [7]. Although the cap-binding residues are located in similar structural elements within the CBD, the number and chemical properties of the interacting amino acids differ between bunyavirus families as well as compared to influenza virus CBD. For instance, while in the influenza virus CBD a classical aromatic sandwich stacks the m7-guanine moiety of the cap, in LACV, the stacking is done by an aromatic and an arginine side chain. The bunyavirus cap-binding pocket also seems to be shallower than that of influenza virus [7,48,49,60,61]. For the arenaviruses, it remains unclear if a cap is indeed bound by the CBD-like domain [48,60]. The composition of the CTER beyond the mid-link domain varies substantially between bunyavirus families (Fig 2). In the LASV L, it resembles the 627-domain of influenza virus PB2 [48], whereas the LACV L contains an α-helical zinc-binding domain (ZBD) [39]. The SFTSV L has a so-called lariat domain that wraps around the CORE and also appears to interact with the ENDO [41,42]. Interestingly, the LACV L ZBD also contains a protrusion in the form of a β-hairpin strut that interacts with the thumb domain of the CORE [39]. Both the β-hairpin strut and the lariat are likely stabilizing the CTER in a particular orientation and are transmitting information to the CORE, potentially playing a crucial role for polymerase activity and the orchestration of conformational changes during viral genome replication and transcription [39].

### 3′ and 5′ promoter binding

**Fig 3:**
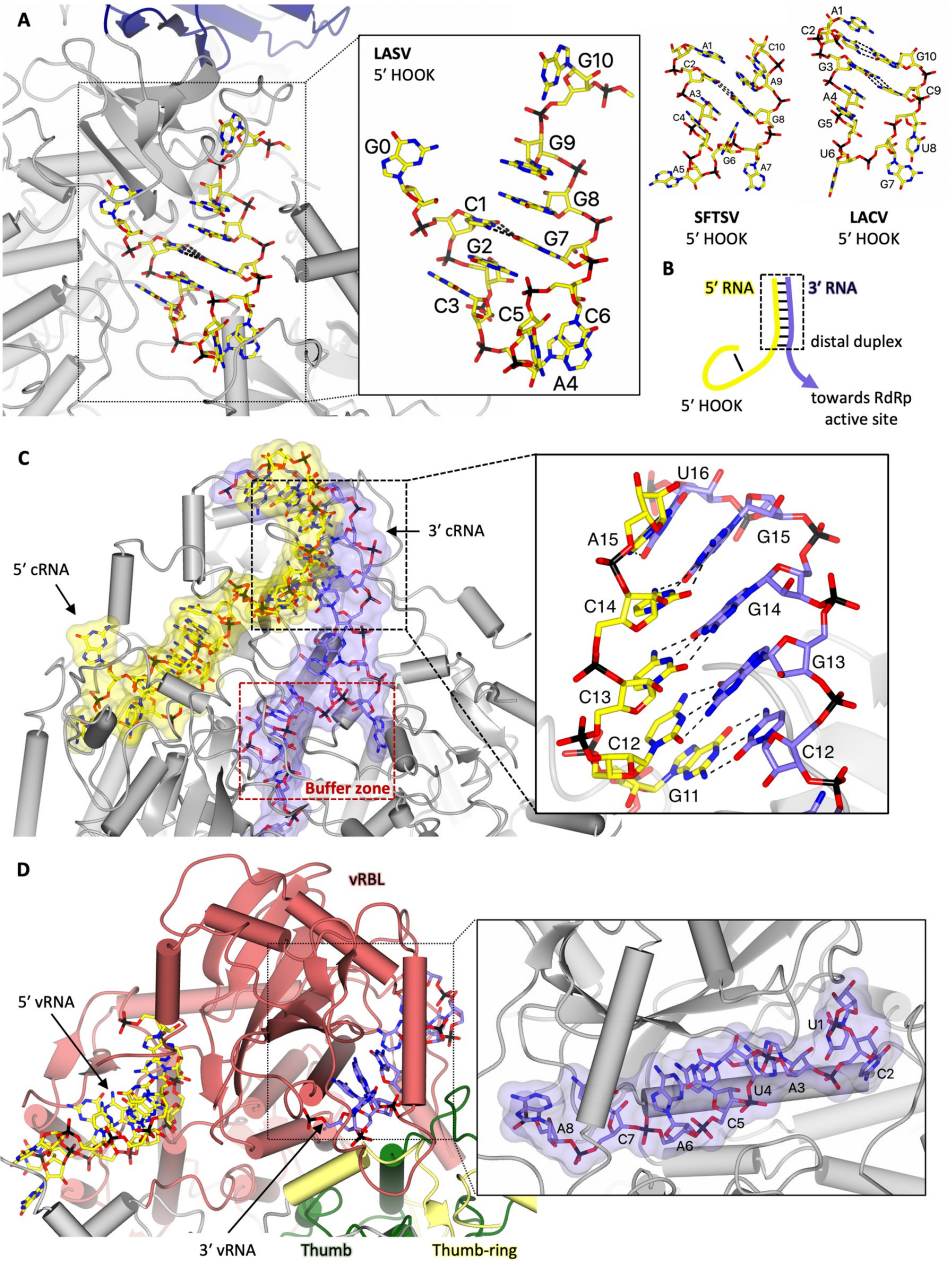
Insights into 5′ RNA hook coordination, distal duplex conformation, and 3′ template RNA sequestration. **(A)** A top-down view of the 5′ vRNA hook binding site in the LASV L protein (PDB: 7OJN) [48] is shown. The L protein core is shown in grey, whereas the pyramid domain is shown in dark blue with the 5′ vRNA coloured yellow. Interactions between cognate base pairs are represented by dashed lines. A close-up on the LASV 5′ vRNA hook is shown with individual nucleotides labelled accordingly. For comparison, the 5′ RNA hook motifs of SFTSV (PDB: 8ASB) [50] and LACV (PDB: 7ORN) [49] are also shown. **(B)** A schematic depiction of the promoter structure at pre-initiation with the 5′ RNA in yellow, the 3′ RNA in purple, and the distal duplex region as well as the 5′ hook labelled. The 3′ RNA would proceed towards the RdRp active site. **(C)** The distal duplex of the SFTSV L early-elongation structure (PDB: 8ASB) [50] is shown demonstrating that the 5′ RNA (yellow) and 3′ RNA (purple) form a corkscrew-like motif that winds around the SFTSV vRBL. The 5′ RNA and 3′ RNA surface is shown at 50% transparency. A close-up of the key base pairs in the distal duplex region is provided revealing that the 5′ cRNA and 3′ cRNA are in fact shifted by 1 nucleotide allowing for more cognate base pair interactions to form. **(D)** A side-on view of the LACV vRBL in the late-elongation structure (PDB: 7ORI) [49] showing the 5′ vRNA bound to the LACV L hook binding site and the tail end of the 3′ vRNA bound to the 3′ secondary binding site is provided. As in (**B**), the 5′ RNA is coloured yellow, the 3′ RNA is coloured purple, and the RNA surface is shown at 50% transparency. A close-up of the 3′ vRNA in the 3′ secondary binding site is shown. Figures were created using CCP4mg [68]. LACV, La Crosse virus; LASV, Lassa virus; RdRp, RNA-dependent RNA polymerase; SFTSV, severe fever with thrombocytopenia syndrome virus; vRBL, vRNA-binding lobe; vRNA, viral RNA cRNA, complementary viral RNA.

Structures of LASV, SFTSV, and LACV L proteins in pre-initiation and early elongation conformations show binding of the conserved genomic 3′ and 5′ RNA termini, which constitute the usually highly complementary and conserved viral promoter, with a length of up to 19 nucleotides [39,48,50]. The 5′ terminal 10 nucleotides bind to bunyaviral L proteins in a structured stem-loop conformation, usually called the hook, which is known to be essential for L protein activation (Fig 3). In LASV, the 5′ hook binds beneath the pyramid domain, whereas in SFTSV and LACV, the 5′ hook binds adjacent to the vRBL [39,48,50].

In LACV L, 5′ hook binding has been shown to result in the ordering of the fingertips region of the RdRp that corresponds to motif F and stabilization of residues surrounding the NTP entrance tunnel [39]. For SFTSV L protein, 5′ hook binding leads to only a partial stabilization of the fingertips region but retraction of a vRBL region from the RdRp active site [50]. Upon template RNA binding to the active site, proper positioning of the fingertips region is observed. In contrast, for the arenaviruses, the fingertips and RdRp active site are well ordered even in absence of vRNA [45,48]. Interestingly, even though some *in vitro* polymerase activity could be observed in the absence of the 5′ RNA, mutations in this binding site resulted in a complete RNA synthesis defect of phenuivirus and arenavirus L proteins in cell-based mini-replicon assays [48,50], which suggests further roles for the 5′ RNA. For the influenza virus polymerase, it was shown that 5′ vRNA hook binding favors a transcription conformation activating the ENDO activity of L protein [69]. The presence, location, and general importance of the 5′ hook binding site seem to be analogous to what has been observed in influenza virus polymerase, suggesting a conserved mechanism for allosteric regulation by the 5′ RNA in sNSV [38,48–50,70,71]. However, not all the mechanisms of the regulatory function of the 5′ hook for bunyavirus L proteins have yet been elucidated.

The 5′ RNA region upstream of nucleotide 10 forms a duplex with the complementary 3′ RNA, denoted the distal duplex (Fig 3). Structurally, in LACV, SFTSV, and LASV L, the distal duplex is important for guiding the 3′ RNA terminus into the active site, where it serves as a template for RNA synthesis. Although for LASV L protein the 3′ terminus is not visible in the active site without the presence of a primer, the trajectory towards the active site from the distal duplex is clearly visible [48]. The presence of the distal duplex leads to stabilization of the pendant and α-bundle in case of LASV L, which are disordered in the absence of vRNA [48]. For the LACV L, the 3′ terminus is visible up to the active site and is compatible with initiation (described in the paragraph below). Its binding leads to ordering of the arch and reorganization of the α-ribbon [39] (Fig 2). For the SFTSV L, in an early elongation state, the distal duplex is still intact and the RNA template could be modelled at full length [50]. Interestingly, in this case, the distal duplex formed by the 3′ and 5′ RNA is shifted against each other by one nucleotide, which results in more base pairs than expected otherwise [50] (Fig 3). These data are supported by mutagenesis studies of the 5′ and 3′ RNA termini in Bunyamwera virus (BUNV, *Peribunyaviridae*) and LASV where the complementarity of nucleotides within the distal duplex was shown to be critical for genome replication and transcription [72–74].

In addition to the expected position of the 3′ RNA towards the active site, a secondary binding site has been observed for LACV, SFTSV, and LASV L (Fig 3). It is formed by the vRBL, or pyramid domain in the case of LASV L, and by the thumb/thumb-ring domains. Upon progression of the polymerase on the template during elongation, the 3′ RNA has been shown to exit the L protein core and bind to the 3′ secondary binding site. This 3′ secondary binding site might therefore act as a protective docking site for the 3′ RNA terminus to enable efficient recycling [49,50]. Mutations of the key interacting residues of L in the secondary site caused a complete defect in RNA synthesis as detected in viral minigenome systems [48,50]. Similarly, any mutations in the terminal 5 residues of the 3′ vRNA entirely abolished activity of the LASV and BUNV L proteins in mini-replicon systems even when they were compensated by complementary mutations in the 5′ terminus [72,73]. For the MACV L, *in vitro* protein–RNA interaction studies led to the conclusion that nucleotides 2 to 5 of the 3′ terminus were essential for template binding, although the existence of a secondary binding site was unknown at that time [75]. Together, these data demonstrate that the base pairing between nucleotides 1 to 5 of the 3′ and 5′ RNA is not important but the specific interaction with the 3′ secondary binding site is [48,73]. Binding of the 3′ RNA to the secondary binding site does not interfere with elongation activity or 5′ hook binding [48,50]. The exact role of the 3′ secondary binding site during genome replication and transcription is, however, not clear yet.

Promoter-bound pre-initiation structures were important to better understand the organization of the L protein. In combination with further structures of functionally relevant conformations, they foster understanding of the molecular mechanisms underlying L protein catalytic activity.

### Viral genome replication

For LACV, SFTSV, and LASV, stalling the L protein in active states for structural determination was linked to the availability or development of functional assays both *in vitro* and in cells. For both LASV and LACV, mini-replicon assays revealed that the tag necessary for protein purification should not be added at the N or C termini as this significantly reduced L protein activity [49,59]. Instead, tags could be inserted internally, for example, in a long, exposed loop called the California insertion within the thumb domain of LACV L [49] or at the tip of the pyramid domain of LASV L [59]. In contrast, C-terminally tagged SFTSV L protein showed robust polymerase activity *in vitro* [40,50]. In the case of LACV L protein, activity assays revealed that mutation of the ENDO active site was crucial to prevent RNA degradation [49]. Additionally, mutation of the 5′ hook to maintain the hook structure and interaction with L but decrease 3′ and 5′ vRNA complementarity was essential for *in vitro* activity [49].

#### Genome replication initiation

**Fig 4:**
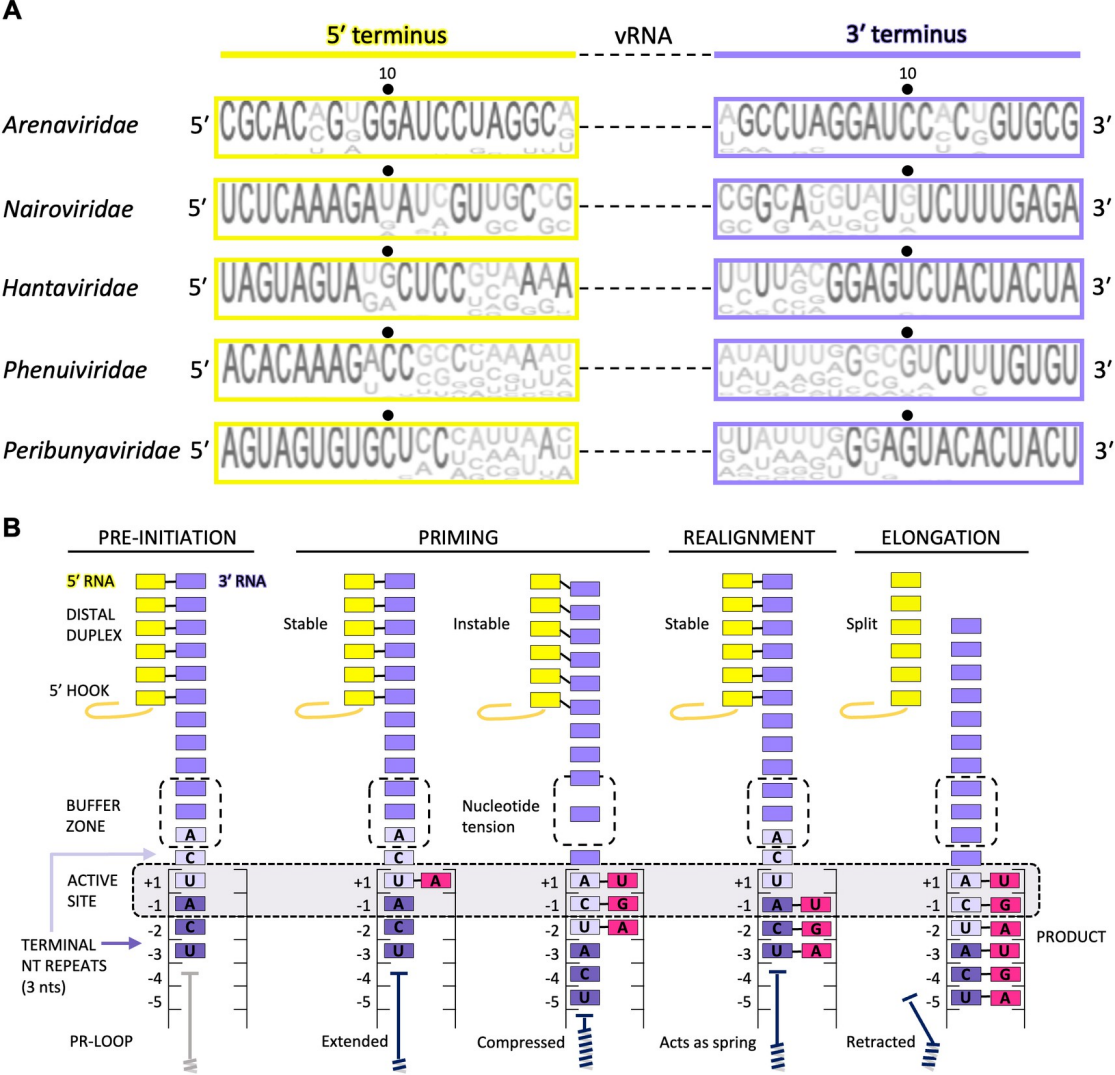
Priming genome replication in the bunyaviruses. **(A)** Analysis of the sequence conservation and nucleotide complementarity in the 5′ and 3′ terminal 20 nucleotides of the vRNA of all genome segments. Sequence conservation was analysed using Jalview [85]. Virus species in the following families were analysed: *Arenaviridae* (*n =* 14), *Nairoviridae* (*n* = 5), *Hantaviridae* (*n* = 14), *Phenuiviridae* (*n* = 14), and *Peribunyaviridae* (*n* = 18), sequences of all genome segments were used. **(B)** Structure-based model of initiation by prime-and-realign for genome replication exemplified for peribunyaviruses. The 5′ RNA is colored yellow and the hook shown as a line. The 3′ RNA is coloured purple with the 3-nt repeats colored light and dark purple. Incorporated nucleotides (product) are coloured in pink. The PR-loop is shown in dark blue except for the pre-initiation panel, where its position is speculative and it is therefore colored in grey. The binding pocket of nucleotides A6, C7, and A8 at priming stage, called buffer zone, is surrounded by a dotted square and labelled. The proposed successive steps (labelled above) of the models are presented from left to right. nt, nucleotide; PR-loop, prime-and-realign loop; vRNA, viral RNA.

Contrary to many viruses that initiate their genome replication and transcription terminally on the 3′ end of the vRNA template, initiation of genome replication by bunyaviral L proteins is thought to occur internally at nucleotide 2, 3, or 4 of the template [76–81]. Internal initiation is also used by influenza virus polymerase for vRNA production from a cRNA template, whereas cRNA production from a vRNA template is initiated terminally [82]. Following internal initiation and *de novo* formation of a 2 to 3 nucleotide primer, which is also the rate-limiting step in the genome replication process, a realignment of the primer to the template terminus takes place. During this realignment step, the template moves backwards in the active site so that the newly aligned primer can be subsequently extended by the polymerase during elongation (Fig 4). This peculiar *de novo* initiation process of RNA synthesis is known as a “prime-and-realign” mechanism and is used to ensure and restore completeness and correctness of the viral genome termini [83,84]. A common feature of bunyavirus genome segments permitting prime-and-realign is the repeat of 2 or 3 nucleotides at the 3′ end (Fig 4).

The details of the prime-and-realign mechanism for initiation of genome replication appear to diverge between bunyavirus families as observed by the differences in the RNA products. In peribunyaviruses, for example, the product has a triphosphorylated 5′ end [86]. It has therefore been suggested that the polymerase initiates RNA synthesis internally at nucleotide +4 of the template (counted from the 3′ end) and generates a 3-nucleotide primer, which is subsequently realigned to nucleotides +1 to +3 of the template prior to RNA elongation. This has now been confirmed by structures of LACV L protein [49]. In the LACV L replication initiation structure, both 5′ and 3′ RNAs are visible with nucleotides 12 to 17 forming a distal duplex and the 5′ nucleotides 1 to 10 bound in a hook-like conformation as observed in the pre-initiation structures [39]. The 3′ nucleotides 1 to 11 enter the RdRp core through the template entry tunnel. The 3′ RNA terminus reaches deep into the active site cavity, going past the active site (+1 position), so that nucleotide +4 of the template aligns with the RdRp catalytic residues where an incoming ATP is also visible, stabilizing the 3′ RNA [49]. This observation further supports a prime-and-realign mechanism for initiation of LACV genome replication. Similarly, in SFTSV early elongation structures, the 3′ RNA terminus reaches past the active site residues into the core with the distal duplex still being intact. Together with further evidence, this observation strongly suggests an internal initiation followed by realignment [50].

**Fig 5:**
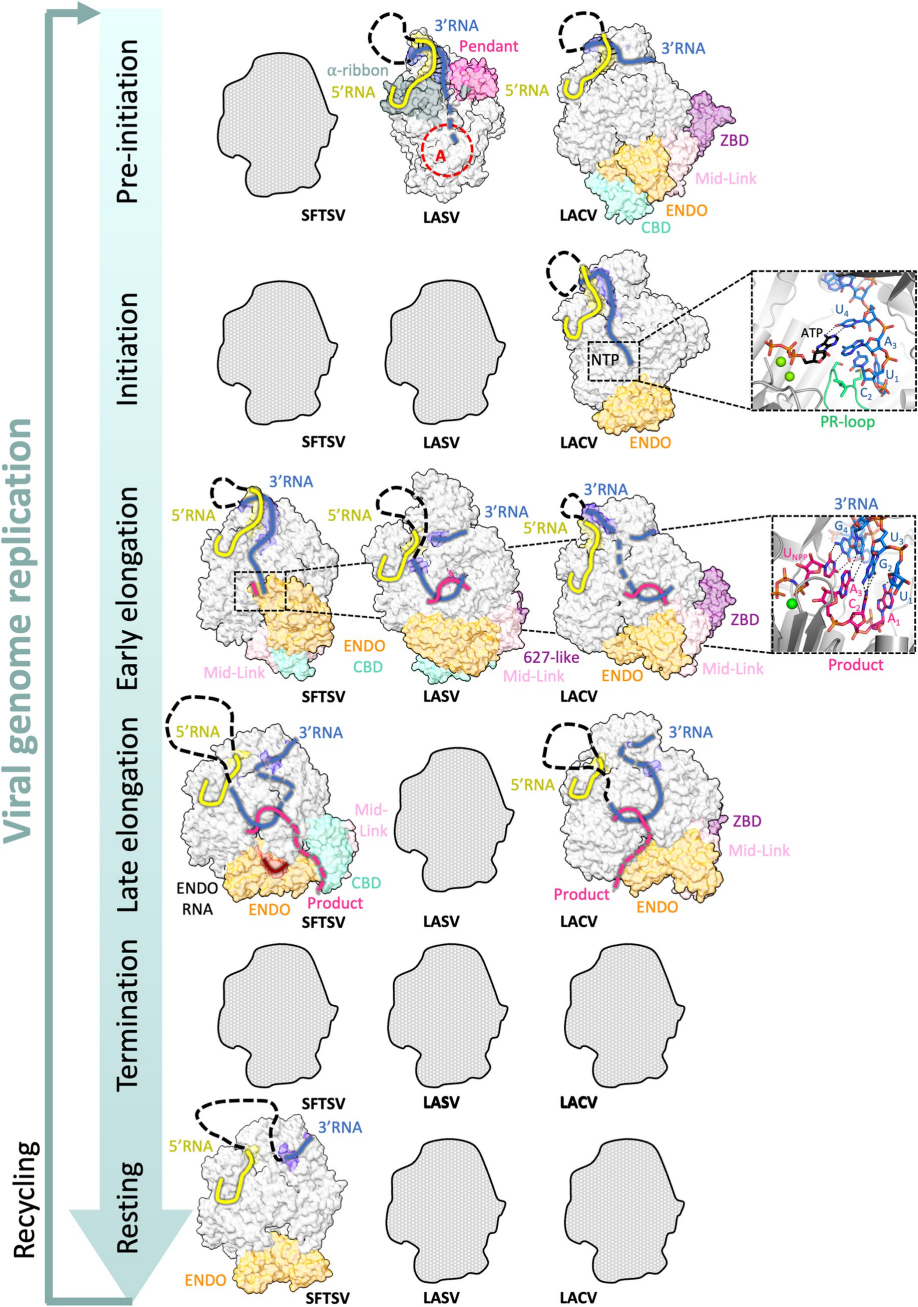
Comparison of bunyavirus L protein structures reflecting viral genome replication. There are several structures of different bunyaviral L proteins that reflect different stages of viral genome replication. For SFTSV, early elongation (PDB: 8ASB), late elongation (PDB: 8ASD), and a resting state (PDB: 8ASG, Ref.) [50] have been visualized. For LASV, pre-initiation (PDB: 7OJL) and early elongation (PDB: 7OJN) [48] have been visualized. For LACV, pre-initiation (PDB: 6Z6G) [39], initiation (PDB: 7ORN), early elongation (PDB: 7ORO), and late elongation (PDB: 7ORI) [49] have been visualized. The RdRp active site is marked by a red dashed circle and labelled with A. Of note, we define late-stage elongation starting when the product-template duplex dissolves allowing the RNAs to exit the inner L protein cavity separately. Protein figures were generated using ChimeraX [92] and Pymol (Schrödinger LLC). CBD, cap-binding domain; ENDO, endonuclease domain; LACV, La Crosse virus; LASV, Lassa virus; PR-loop, prime-and-realign loop; RdRp, RNA-dependent RNA polymerase; SFTSV, severe fever with thrombocytopenia syndrome virus; ZBD, zinc-binding domain.

For some RdRps that perform terminal initiation of *de novo* RNA synthesis, a structural element called the priming loop inserts close to the active site to stabilize the initiation complex comprising the first two NTPs (“priming” and “incoming”) [46,87–89]. For the influenza virus polymerase, the priming loop has been shown to be critical for terminal initiation of vRNA to cRNA replication but less so for internal initiation of cRNA to vRNA replication [89]. For LACV, structural data suggest that the putative priming loop element of LACV L is located too far away from the active site to support this role [49], consistent with absence of terminal initiation. Instead, the structure reveals that another loop, denoted the prime-and-realign loop (PR-loop), extends at the initiation state to stabilize the 3′ template end inside the active site cavity (Fig 5). This loop connects the conserved fingers and palm domains and is thus also present in the other sNSV polymerases for which structures have been determined so far, but always in a more retracted conformation as visible in LACV L at elongation state [38–45,48–50]. Functional studies of the LACV L with mutations to the PR-loop support its role in stabilizing the template at initiation and in promoting realignment [49]. The sequence of the PR-loop is not conserved in other sNSV, except for the hantavirus L protein, where some residues show conservative mutations or even are identical [49]. However, it remains to be seen if the PR-loop has the same role in hantavirus genome replication initiation. Analysis of the LACV L initiation structure led to a hypothesis on how realignment could occur following internal initiation. During initiation of genome replication, the 3′ template RNA is stabilized by the distal duplex on one side and the PR-loop on the other side (Fig 4). A buffer zone around nucleotides 6 to 8 is observed in which the nucleotides are less coordinated, and it is conceivable that in this zone extension or compaction of the RNA could happen. The translocation of the 3′ RNA upon RNA synthesis could create tension between the distal duplex and the PR-loop, which could then be discharged in a spring-like mechanism by which the template slips backwards and the initial 3-nucleotide product gets realigned to the terminus of the 3′ RNA template [49].

The arenavirus genome 5′ end contains an additional triphosphorylated G [78]. It was hypothesized that in this case, initiation starts at nucleotide +2 of the template producing a pppGC primer, which is then realigned to the template 3′ terminus (positions −1 and +1) generating this 5′ G overhang. Interestingly, it was shown that the presence of this extra G enhanced viral polymerase activity [59], which is in line with structural data showing a base-specific recognition of the extra G in the 5′ hook binding site [48]. In addition, a potential duplex of the highly complementary 3′ and 5′ RNA with an extra G overhang at the 5′ does not trigger antiviral defense measures for arenaviruses via host cell pattern recognition receptors [90], contrary to what has been shown for phenuiviruses and peribunyaviruses [86]. Triphosphorylated single-nucleotide 5′ overhangs in dsRNA have been shown to even act as decoys for RIG-I, thereby counteracting antiviral signaling [91].

In hantaviruses, the product of genome replication is monophosphorylated at the 5′ end. It has been suggested that initiation starts internally at nucleotide +3 of the template with an initial GTP generating a short primer [81]. This short primer would then be realigned to the 3′ terminus, resulting in an extra pppG overhang at the 5′ end of the product, which is subsequently removed by a putative nuclease resulting in a monophosphate at the 5′ end. The mechanism behind this cleavage remains to be determined.

For phenuiviruses, particularly SFTSV, both terminal initiation and internal initiation at position +3 with subsequent realignment of a dinucleotide could be equally well explained by biochemical data [40]. In both scenarios, no 5′ overhang would be generated. More recent structural data for SFTSV L suggest a similar mechanism as for LACV L. One important observation supporting this hypothesis was that in an early elongation structure, the template reached deep into the active site with the distal duplex still intact. Also, an RNA region similar to the so-called buffer zone in LACV is visible (Fig 3). However, in SFTSV, the prime-and-realign mechanism is potentially independent of a PR-loop, the equivalent of which is not conserved in sequence compared to LACV L [50].

Overall, structural data of genome replication at the initiation stage are still rare, likely due to the transient nature of these conformations. Therefore, although there have been many hypotheses about the presence and location of priming loops within the L proteins of LASV, JUNV, SFTSV, and RVFV, all of these remain highly speculative for now [41,44,45].

#### Genome replication elongation

Structures of L proteins stalled at elongation have been determined for LASV, LACV, and SFTSV [39,48,50]. LACV and SFTSV L exhibit a 10-bp template-product duplex in the active site cavity, whereas for LASV L, only an 8-bp duplex has been observed so far. Conformational changes between initiation and elongation states in LACV L include (i) the retraction of the PR-loop to provide space for the template-product extension. This is coupled with (ii) a large movement of the ENDO involved in stabilizing the extended PR-loop conformation. (iii) Concomitantly, the CORE opens to accommodate the template/product duplex with concerted movement of the thumb, the thumb ring, and the lid. (iv) The loop initially thought to be a putative priming loop is extruded from the template exit tunnel, as the template translocates. Therefore, this loop has been renamed the template exit plug to better describe its role in LACV L. This opening up of the active site is also visible in SFTSV L, whereas for LASV L, it is less obvious, possibly because the initiation to elongation transition is incomplete. Overall, the major changes upon elongation occur in the accessory domains of L (Fig 5). The ENDO in SFTSV seems to be quite mobile at early elongation and can adopt an alternative conformation. In the elongation structure of LASV L, the CTER region is stabilized in a ring-like form around the putative product exit channel and interacts with the ENDO and CORE regions. In addition, the LASV ENDO was found to be autoinhibited during elongation. Late-stage elongation structures of LACV and SFTSV L using a longer 3′ RNA template showed that, following its exit from the RdRp active site cavity, the template progresses along a positively charged groove that leads to the 3′ secondary binding site, as first shown for influenza virus polymerase [47].

#### Genome replication termination

To date, there are no structural data visualizing chain termination in bunyavirus genome replication. If it is not provided in *trans*, the 5′ end must be released from its dedicated binding site to pass the active site and allow for production of a full-length cRNA or vRNA. After product disassociation, one can speculate that the template backtracks so that the 5′ end rebinds to the 5′ binding site in the same L molecule, but there are no structural data published supporting this hypothesis yet. Notably, in case of arenaviruses, the nontemplated extra G nucleotide added to the 5′ terminus during prime-and-realign is not replicated and therefore does not lead to the addition of an extra C at the 3′ end. RNA synthesis is therefore terminated in a way that retains the original 3′ sequence. The genome segments packaged into nascent virions, therefore, contain only a single-nucleotide G overhang at the 5′ end [78].

### Viral transcription

#### Cap-snatching

Viral transcription is initiated by a primer, a capped host RNA fragment obtained by cap-snatching. In the case of influenza virus, cap-snatching occurs in the nucleus and is rather well understood, while for the cytoplasmically replicating bunyaviruses, many aspects, such as the nature, source, and location of the target capped RNA, remain obscure as reviewed previously [7]. However, very recent structural data have added to our understanding of cap-snatching in different bunyavirus families. Fundamentally, the L protein needs a CBD to bind to the 5′ cap of the host mRNA and an ENDO to cleave the RNA. While the presence of these two functions has been clearly demonstrated for some L proteins, such as SFTSV, RVFV, and LACV L proteins [40,49,61,93–96], the functionality in other L proteins, in particular, arenaviruses, hantaviruses, and nairoviruses, is less clear, even if the domains are present [7,48,60,97–99]. In contrast to the hantavirus ENDO, which seems to be highly active, even limiting its own recombinant expression in cells [99–101], the arenavirus and nairovirus ENDO is poorly active or inactive *in vitro*, although their essential role in viral transcription was detected in cell-based studies [48,60,98,102–106].

Structurally, all ENDOs have a similar two-lobed, kidney-shaped architecture, harbor a PD(E/D)K active site motif, and coordinate two divalent metal ions essential for catalytic activity [7,60,93,95,96,99,101,103,104]. The ENDOs that have been found to be active *in vitro* possess a catalytically important histidine residue upstream of the PD(E/D)K motif, which is implicated in first cation coordination, whereas the ENDOs with very low or absent *in vitro* activity contain a glutamate or aspartate residue instead. Structural knowledge was initially obtained via X-ray crystallography studies of isolated ENDO domains from LACV (*Peribunyaviridae*), Lymphocytic choriomeningitis virus, LASV, Pichinde virus (PDB-ID: 4i1t), California Academy of Sciences virus (CASV) (*Arenaviridae*), Hantaan virus, Andes virus (*Hantaviridae*) as well as Toscana virus and SFTSV (*Phenuiviridae*) [60,93,95,96,99,101,103,104]. Interestingly, in cryo-EM structures of the full-length LASV L protein, two different mechanisms of ENDO autoinhibition are observed [48]. In the absence of vRNA, a loop of the LASV L protein CORE region (amino acids 1092 to 1105) binds to and blocks the ENDO active site. During RNA elongation, the ENDO residues 181 to 188 of LASV L rearrange and block the active site. For SFTSV L, blocking of the ENDO active site has been observed during early elongation by residues 211 to 233 of the ENDO linker domain, which is facilitated by a repositioning of the ENDO [50].

The global fold of the CBD and CBD-like domains (determined either isolated or in the context of the full-length L) is conserved. It is composed of a β-sheet that packs against a conserved α-helix, and additional elements that vary between families. The cap-binding residues are located on the first β-strand of the central β-sheet and either in a β-hairpin (for RVFV, SFTSV, and influenza virus) or a loop (for LACV) connecting two strands of the β-sheet. The CBD has been solved in complex with capped RNA for LACV, and with m^7^GTP for SFTSV and RVFV [40,49,61]. CBD-like domains of LASV and CASV L were solved in the absence of the cap-ligand [48,60]. The CASV L CBD-like domain appears minimalistic and may not contain any cap-binding site. By comparison, the LASV CBD-like domain is larger than other known CBD structures, has an elongated form, and despite mutational evidence for the importance of the CTER in viral transcription, it remains unclear if this domain harbors a cap-binding activity [48,97]. It therefore remains to be determined if the arenavirus CBD-like domain is indeed functional.

Despite the global similarities, the structure of LACV CBD was somewhat surprising as it does not bind the cap mainly via aromatic stacking as observed in many other cap-binding proteins [7,40,61,107–109]. Instead, it stacks the cap-moiety between an aromatic and an arginine sidechain. This further emphasizes the lack of a sequence motif for cap binding and even structurally this function can be accommodated by different local clusters of amino acids [110]. Interestingly, in the *apo* structure of SFTSV L protein, the cap-binding site was found to be blocked by an arginine sidechain stacking between the two aromatic sidechains responsible for cap-binding revealing a potential regulatory mechanism of cap-snatching [41–43].

**Fig 6:**
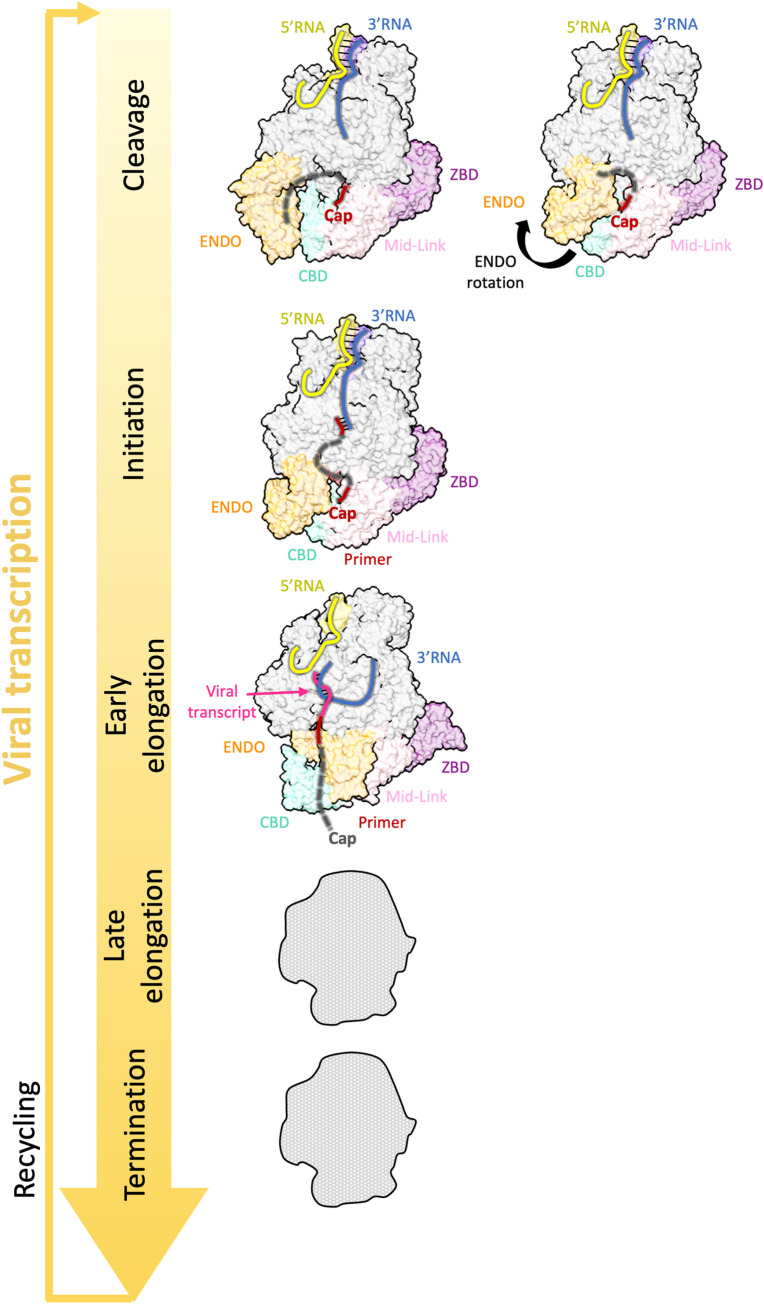
Comparison of bunyavirus L protein structures reflecting viral transcription. The structural insights into bunyaviral transcription are limited to LACV L protein structures [49]. LACV L protein has been visualized during capped primer cleavage (PDB: 7ORJ), followed by the rotation of the ENDO for subsequent primer entry (PDB: 7ORK). In addition, the initiation (PDB: 7ORL) and early-elongation (PDB: 7ORM) steps could be visualized. Protein figures were generated using ChimeraX [92]. CBD, cap-binding domain; ENDO, endonuclease domain; LACV, La Crosse virus; ZBD, zinc-binding domain.

The structure of LACV L has been determined in three different conformations related to viral transcription, respectively called “Capped primer cleavage,” “initiation,” and “early elongation” [49] (Fig 6).

#### Capped primer cleavage

The capped primer cleavage conformation is suggested to be compatible with cleavage of the capped RNA by the ENDO during cap-snatching. Compared to the pre-initiation conformation, the capped primer cleavage structure reveals a large conformational change of the CTER and the ENDO together with a 20° rotation of the ZBD towards the CORE generating a closed conformation of the entire LACV L protein. A concerted movement of the CBD brings the cap-binding site, which was exposed to the solvent in the pre-initiation state, to a more buried conformation, where it interacts with the CORE. Although only the first two residues of the capped RNA are resolved in this structure, it is conceivable that the RNA projects towards the ENDO active site as the path is open. So far, no structure of LACV L with RNA bound to the ENDO has been published. Although this has been achieved for SFTSV L, transcription state structures of the phenuivirus L are still missing [50].

#### Transcription initiation

Following cleavage, the capped RNA primer needs to be reoriented towards the RdRp active site. It was suggested that for the LACV L, primer RNA entry into the RdRp active site cavity results from the electrostatic repulsion of the ENDO, which reorientates between the cleavage and initiation conformation [49]. In the transcription initiation state, the capped RNA is well coordinated at both extremities by the CBD on one side and the RdRp active site cavity on the other side with a few nucleotides unresolved in the middle of the RNA [49] (Fig 6). This is consistent with the fact that diverse RNA sequences and lengths can be bound and used as capped RNA primers [7].

Prime-and-realign has also been observed in transcription of sNSVs [111–116]. Whereas for peribunyaviruses and hantaviruses this results in the addition of one or several nucleotide triplets, in phenuiviruses and arenaviruses, it results in addition of dinucleotides between the capped host RNA primer and the RNA sequence complementary to the template. It has been suggested that the length of the host RNA primer may influence the number of successive realignments. Transcription elongation without realignment was suggested to occur with relatively long host RNA leaders, whereas incorporation of additional triplets was detected for short host RNA leaders [113].

In the transcription initiation structure of LACV L protein, capped RNA entry into the active site cavity is correlated with the retraction of the PR-loop that stabilizes the 3′ template in the cleavage conformation [49]. During transcription initiation, the nucleotides 13 and 14 of the capped RNA primer align with nucleotides 1 and 2 of the 3′ RNA template [49]. In that particular structure, the initiation complex has been stalled at position 4 of the template with the distal duplex being still intact and allowing for additional stabilization of the template RNA [49]. Upon addition of another nucleotide to the transcription product, the template would be pulled further into the active site cavity, which would destabilize the distal duplex and likely generate some tension in the buffer zone. At that point, two scenarios are conceivable: (i) the PR-loop retracts completely, the distal duplex breaks, and the polymerase switches to elongation mode; or (ii) one or several rounds of prime-and-realign occur mechanistically similar as described for genome replication. Both scenarios are supported by the structural and functional data that show importance of the PR-loop for the formation of both realigned and non-realigned products *in vitro* [49].

#### Transcription elongation and termination

Following initiation, the L protein proceeds to elongation. In the LACV L transcription elongation structure, this is correlated with movements of the CBD and the ENDO back to the position they had at pre-initiation stage [49] (compare Figs 5 and 6). The transition is also coupled to an opening of the CORE as described for genome replication. After product-template duplex separation, the product exits the RdRp active site cavity staying close to the ENDO but remaining distant from the ENDO active site. The cap extremity is not visible in this structure as it is detached from the CBD [49] (Fig 6).

Different to cellular mRNA and influenza virus transcription, bunyavirus transcription products are usually not polyadenylated, the only exception being hantaviruses, in which mRNAs transcribed from the M genome segment terminate in a poly-U stretch and therefore contain a poly-A sequence [117]. Most RNA transcripts are terminated at specific positions downstream of the viral genes but before the 5′ terminus of the template, with only some RNA transcripts extending until the end of the template [76,118–123]. Termination at specific positions has been linked to the presence of specific termination signals, likely in form of RNA secondary structures, that differ between viral families, although similar signals have been detected in peribunyaviruses and phenuiviruses [123]. As for termination of genome replication, the structural basis for termination of viral transcription remains to be elucidated.

### The role of additional viral and host proteins

Given that the L protein produces different products (mRNA and cRNA / mRNA and vRNA) from the same template, there must be some kinds of regulatory mechanisms in place to control the generation of these different RNA species. Notably, many bunyaviruses use the vRNA as a template for viral genome replication and transcription but prefer genome replication in presence of the cRNA as a template. For BUNV, it has been reported that the sequence of the 3′ RNA terminus is crucial for this preference but not the only determinant [124]. On the protein level, one aspect of regulation might be dimerization of L protein as shown to be essential for the two senses of influenza virus replication (i.e., vRNA and cRNA synthesis) [125], but not for transcription. Indeed, two different influenza virus polymerase dimers have been implicated in replication, one symmetric dimer, thought to be involved in realignment in early vRNA to cRNA synthesis [126] and a second asymmetric “replicase-encapsidase” dimer, which is stabilized by the host protein ANP32 (acidic nuclear protein 32) [127] and thought to promote recruitment of NP to the product and its packaging into a progeny ribonucleoprotein complex (RNP) [128]. Thus, replication can only occur after accumulation of sufficient quantities of newly synthesized polymerase and NP. Various L protein dimers have also been observed for LASV and MACV [45,52], and L–L interaction has been suggested to be necessary for arenavirus L protein RdRp activity [129–131]. This would fit with the observation that tag insertion at the C terminus of LASV L reduces protein dimerization *in vitro* and reduces RdRp activity in a cell-based minigenome system [59]. However, the dimers observed for arenavirus L proteins so far have been all symmetric [45,52,127].

Another regulatory switch occurs when arenavirus Z protein binds to the L protein. Z has been shown to inhibit the L protein RdRp activity in both *in vitro* and cell-based assays [54,56–59,132]. Direct interaction has been suggested based on co-immunoprecipitation experiments [56,133] and electrophoretic mobility shift assays [58] in the past and could be demonstrated recently by cryo-EM structures of L-Z complexes published for three different arenaviruses but in the absence of vRNA in the RdRp active site [51–53]. The mechanism of Z-induced RdRp inhibition is, however, not entirely clear yet. Alternative hypotheses include limited flexibility of the RdRp active site residues upon Z binding, blocking of the product exit channel, and inhibition of the functional elongation conformation due to steric clashes with the C-terminal 627-like domain [48,51–53]. As the L-Z complex structures did not contain vRNA in the RdRp active site, it was not structurally proven that Z protein binding indeed locks the L protein-RNA complex as suggested by biochemical studies [58]. Considering the biological importance of this interaction, due to the ambisense coding strategy of arenaviruses, the Z protein is transcribed from the cRNA, which in turn requires prior genome replication. As a matrix protein, Z is also important for particle budding [134,135]. The inhibition of L protein activity, therefore, fits with the late phase of infection, where a transition from active mRNA and genome synthesis to genome packaging is expected. Notably, while other bunyavirus families do not contain a Z protein, the cytoplasmic domain of their glycoprotein Gn contains a zinc-binding motif [136], a late domain motif commonly involved in viral budding [137,138], and both Gn and also Gc interact with the viral RNPs, consisting of vRNA, NP, and L [139,140]. The cytoplasmic domains of Gn and Gc likely act as Z protein surrogates in other bunyavirus families [138]. An additional exception is the genus hartmanivirus within the *Arenaviridae* family, which was found to not contain any Z protein or similar. It is unclear which protein drives budding and RNP recruitment in this case [141].

Structurally, the bunyaviral RNP, the functional unit of genome transcription and replication, is a complex of vRNA or cRNA encapsidated by the NP and associated with L. The lack of structural data exploring how these RNPs, firstly, scaffold the processes of genome replication and transcription and, secondly, are coreplicationally assembled is one of the major outstanding problems for bunyavirus research. However, although at low resolution, recent studies looking at isolated RNPs of BUNV and MACV have provided us with important new insights into these complexes [142,143]. For MACV, negative-stain EM images of purified vRNPs demonstrate that they adopt relatively diverse and flexible pseudo-circularized structures [143]. Curiously, the EM data seem to suggest that there are multiple L proteins associated with each NP-RNA chain rather than a single L protein localized to the 5′ and 3′ termini [143]. In the study on BUNV RNPs, the purified RNPs mainly lack the L protein, potentially lost during RNP isolation, while still retaining a circular shape [142]. These two studies both disagree with one of the two prevailing theories on how bunyaviral RNPs are structured, namely the “beads on a string” model, which was based on early EM data showing RNPs with a diameter of a single NP [20,23]. Instead, the observed MACV RNPs and BUNV NP-RNA structures support the alternative model in which RNPs adopt a relaxed and helical architecture, with the RNA encapsidating NP monomers forming a flexible helical structure [21,24]. However, artifacts from RNP isolation affecting the overall structure cannot be excluded. The influence of ionic strength and pH on the structure of viral RNPs has been demonstrated, for example, for vesicular stomatitis virus [144].

It is clear that L and NP need to interact during genome replication and transcription. Firstly, for both processes, NP needs to be stripped from the template as it enters the polymerase active site and the NP needs to be rebound to the outgoing template to preserve RNP integrity. Secondly, for replication only, the nascent vRNA and cRNA are packaged by newly synthesized NP, whereas transcripts are not packaged. It is plausible, therefore, that NP plays a regulatory role in balancing viral transcription and genome replication [145], although the exact mechanisms are not clear. Indeed, it was shown for lymphocytic choriomeningitis virus that the intracellular concentration of NP does not influence the transcription-to-replication ratio [146]. In addition to viral proteins and RNA, host proteins and RNA might be also important factors for genome replication and transcription processes as well as their regulation. A coupling of viral transcription and mRNA translation was demonstrated for peribunyaviruses [147]. Diverse and complex interaction networks have been described for both NP and L proteins [148–152]. Although no mechanistic insights have yet emerged on transcription and replication regulation, it is likely that host factors may influence these processes by recruiting RNPs to specific microenvironments within the cell (for instance, where capped RNAs are present) or by facilitating posttranslational modifications that may be essential for certain functions. Maybe, and this is purely speculative, in transcription-related microenvironments, repackaging of the template is not even necessary, therefore independent of NP concentrations [146], and instead of the template passing the active site entirely as expected for genome replication, it may simply slip backwards in the L protein to recycle to pre-initiation state. In addition, the role of posttranslational modifications of L and NP in bunyaviruses is very poorly understood. Therefore, additional data are needed before any solid conclusions can be made on the role of host proteins during genome replication and transcription.

### Comparison of bunyaviral L proteins with orthomyxovirus polymerases

**Fig 7:**
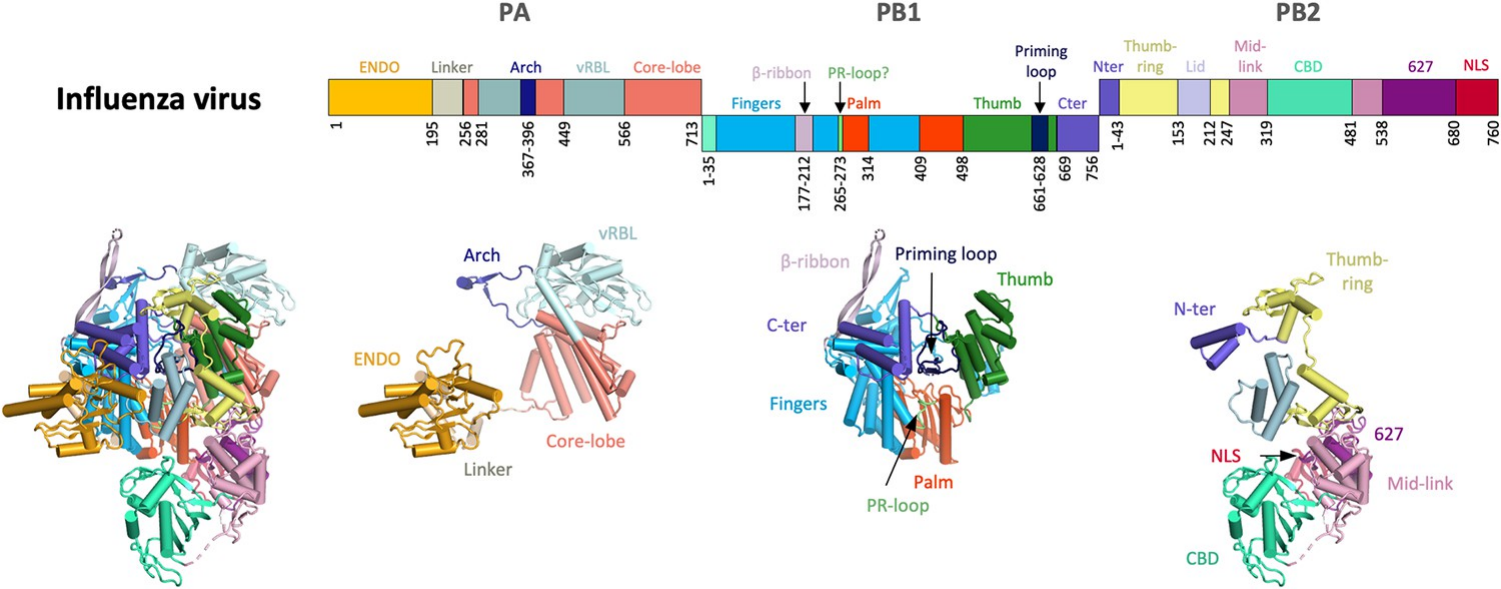
Structure of influenza virus polymerase complex for comparison. The influenza virus polymerase complex (PDB: 4WSB) is presented as a schematic linear representation with the size of the individual domains scaled to represent their relative size in the trimeric polymerase complex. Individual domains are labelled and colored. The colors for each domain are mapped onto a cartoon representation of the trimeric complex, which is then broken down into the subunits PA, PB1, and PB2. Figures were created using Pymol (Schrödinger LLC). CBD, cap-binding domain; ENDO, endonuclease domain; NLS, nuclear localisation signal; PR-loop, prime-and-realign loop; vRBL, vRNA-binding lobe 627, 627-like domain, Cter, C terminus, Nter, N terminus.

Due to its perennial threat to global public health through seasonal epidemics and periodic pandemics, the influenza orthomyxovirus has received considerably more attention than other segmented RNA viruses such as bunyaviruses, which tend to cause much more localized outbreaks. This also extends to detailed understanding of the transcription and replication mechanisms involving the polymerase. It is therefore interesting to compare the heterotrimeric orthomyxovirus polymerase complex to the monomeric bunyavirus L proteins and identify common and distinct features. Although these viruses are closely related, the location of genome replication and transcription inside the infected cell is distinct with the orthomyxovirus polymerase complex localizing to the nucleus, whereas bunyavirus L protein remains in the cytoplasm. However, it is now well established that the overall architecture of the monomeric L protein corresponds to a concatenation of the three influenza virus polymerase subunits in the order PA-PB1-PB2 [38,93] (compare Figs 2 and 7).

In all segmented RNA viral polymerases, the mobile ENDO and CBD coordinate cap-snatching. Unlike for bunyaviruses, the source of, and mechanism of access to, the capped primers required for transcription is known for influenza virus. In the nucleus, cap-snatching occurs through the binding of the influenza virus polymerase to the Serine 5 phosphorylated CTD of initiating cellular Pol II giving access to nascent capped transcripts [153–155]. Concerning RNA promoter binding, both influenza and bunyaviruses exhibit a 5′ hook binding site as well as a 3′ secondary binding site in their polymerase, although the detailed secondary structure of the 5′ hook itself slightly varies [50]. Both RNA binding sites are in the same relative position. Mutations to the 3′ secondary binding site resulted in complete loss of polymerase activity in viral mini-replicon systems for influenza virus, SFTSV, and LASV [48,50,156]. At pre-initiation, all polymerases also contain a distal duplex in the promoter with the single-stranded 3′ end of the template capable of entering the polymerase active site or 3′ secondary binding site. The conserved nature and positioning of these RNA binding sites implies that the template trajectory is also conserved, likely both for transcription and replication, with the template exit channel directly leading into the 3′ secondary binding site [47]. Docking of the template 3′ end in this site forces the template to bulge out as RNA synthesis proceeds providing a plausible mechanism for preservation of the vRNP structure, involving NP transfer across from the incoming template to the outgoing template and efficient recycling, as elucidated for influenza virus polymerase [47].

Significant structural differences between influenza virus and bunyavirus polymerases mainly lie in the idiosyncratic domains in the CTER beyond the mid-link and CBD. In influenza virus polymerase, these are the 627 and NLS (nuclear localisation signal) domains. The 627 domain is critical for mediating host factor ANP32-dependent asymmetric dimerisation of the polymerase during genome replication, and the NLS enables nuclear import of the PB2 subunit through interaction with importin-α. Since they interact with host proteins, both these domains contain important host-specific amino acid variations (for instance, dependent on whether the host is avian or mammalian). In LASV, there is also a 627-like domain but with a distinct extreme CTER. In LACV and SFTSV, this region contains unique structures, respectively, a ZBD with a protruding β-hairpin strut and a loop-like lariat, both of which bridge to the CORE, stabilising the whole CTER in certain functional states. Unfortunately, to date, there is no knowledge about how any host factor interacts with L proteins. Another important difference stems from the fact that the two single-stranded ends of the promoter are less complementary and crucially of different lengths in influenza virus when compared to bunyaviruses. A consequence of this is that for influenza virus polymerase, *de novo* initiation happens terminally for vRNA to cRNA replication, and internally for cRNA to vRNA replication [82], since the longer cRNA 3′ end extends further into the active site. In bunyaviruses, the 3′ and 5′ ends are the same length and all replication initiation seems to be performed internally by prime and realign. Perhaps for this reason, only the influenza virus polymerase has a true priming loop, which is required to stabilize the terminal initiation complex with two incoming NTPs aligning with the first two nucleotides of the template [89]. No obvious priming loop has been identified in any bunyavirus L protein so far. The PR-loop identified in LACV L (see above) is not an equivalent of the priming loop in influenza virus, and it remains to be seen how this works in other systems.

Another difference between influenza viruses and bunyaviruses is the relative position of the CORE, ENDO, and CBD at transcription. In the influenza virus polymerase complex, the CBD undergoes a large rotation to first bring the capped RNA towards the ENDO for cleavage and subsequently insert it into the RdRp active site for transcription priming [70]. This differs from LACV L, which displays large rearrangements of both ENDO and CBD between the pre-initiation and the capped-RNA cleavage conformation [49]. Movement of the capped RNA from the suggested cleavage conformation to the initiation is then triggered by a large ENDO rotation. Although in the SFTSV L *apo* structure, the ENDO and CBD were visible and their position opposite compared to the influenza virus polymerase [41], the transcription-related positions and movements of these domains need to be determined before any mechanistic conclusions can be drawn.

In some bunyaviruses, the ENDO and CBD have been observed to be self-inhibited, which is not the case for influenza virus (although access to their functional sites can be occluded). For LASV, two different examples of self-inhibition of the ENDO by other peptides of the L protein have been observed, and for SFTSV, the CBD is self-inhibited by an arginine (R1843) from an adjacent L protein region, necessitating a conformational change to allow capped primer binding. Finally, there are key functional differences between mechanisms of termination of influenza virus polymerase and bunyavirus L proteins. Whereas during transcription termination, influenza virus polymerase polyadenylates the viral mRNA by stuttering, the mechanism of which is now rather well understood [47], for most bunyaviral L proteins, there is no polyadenylation and the mechanisms of internal (during transcription) and terminal (during replication) termination are currently not known.

## Conclusions

In recent years, tremendous progress has been made in characterizing the structure and function of the L protein, which, in turn, has given new insight into the detailed mechanisms of bunyavirus genome replication and transcription. These new structural data open a new era in bunyavirus research where functional studies on genome replication and transcription can be supported by structural data or structure predictions. However, many gaps need to be filled to achieve a complete picture of these processes and their regulation. What is the role of L protein dimers? What determines the different modes of termination during transcription and replication? How is the 5′ RNA released from its dedicated binding pocked during genome replication? How do the different primer lengths detected in bunyavirus families [7] relate to the structures of L proteins during transcription? What is the role of host proteins in, for instance, cap-snatching, RNP packaging, and transcription-translation coupling?

Although bunyaviruses have a relatively simple composition, their proteins and RNA are multifunctional, multifaceted, and depend not just on each other but also on host factors. Dissecting the exact and potentially multiple roles of each functional element within the RNA and proteins will allow for comparisons to be made and commonalities to be identified between bunyavirus families. Although this will be challenging, it is essential and will allow for the rational design of antiviral strategies. Looking at the progress made on related viruses, we expect the next major step to be the determination of the structure and dynamics of functional RNPs and modelling of their assembly and structural changes during genome replication and transcription processes. Discovery of host factors involved in these processes will be instrumental to obtain a full picture. Maybe electron tomography will allow us to visualize and understand these processes in the context of the cell. We therefore look forward to even more exciting research on these highly relevant viruses.
